# Mineralogical variations of sand sediments in the Tigris and Euphrates Rivers: implications for agricultural sustainability

**DOI:** 10.1007/s10661-025-14407-6

**Published:** 2025-07-23

**Authors:** Layth Saleem Salman Al-Shihmani, Ahmed Abed Gatea Al-shammary, Mahdi Wasmey Seheib Alaidi, Jesús Fernández-Gálvez, Andrés Caballero-Calvo

**Affiliations:** 1 Department of Soil and Water Science, College of Agriculture, University of Wasit, Kut, Iraq; 2 Department of Regional Geographical Analysis and Physical Geography, University of Granada, Granada, Spain

**Keywords:** Sand sediments, Tigris and Euphrates Rivers, Agricultural sustainability

## Abstract

Climate change and human activity have impacted the quantitative and qualitative characteristics of sediment content in Iraq’s Tigris and Euphrates rivers. This study aims to determine the spatial variation of sediment characteristics, including sand mineral content and degree of maturity, in the two rivers. This study highlights the importance of sediments for agricultural soils and plants, emphasising their role in enhancing soil properties when deposited naturally or added by humans. It evaluates the chemical and physical characteristics of riverbed sediments, with a particular focus on identifying the mineral composition of both heavy and light sand minerals. Furthermore, it examines their mineral maturity. Chemical tests revealed an increase in the electrical conductivity downstream in both rivers. Physical assessments indicate a downstream decrease in the proportion of sand particles and a corresponding increase in the proportion of clay particles. Light sand minerals constitute a significant portion, ranging from 95.6 to 96.8% of the total mineral content, encompassing diverse minerals such as quartz, feldspar, and fragmented rock minerals. Heavy sand minerals account for between 3.2 and 4.4% and include opaques, chlorite, pyroxenes, hornblende, mica, zircon, tourmaline, and garnet. This trend shows a decrease in the overall maturity of these sediments, with a maturity trend shifting towards physical maturity. In contrast, the maturity index of light sand minerals decreases with increasing distance travelled along the two rivers. Understanding these mineralogical variations provides insights into the intricate interplay of geological, climatic, and anthropogenic factors shaping river-loaded sediments. This knowledge helps in choosing sustainable agricultural practices for soils to which these sediments are added.

## Introduction

Studies on the mineral composition of sand minerals in river sediment are fundamental for identifying the types of source rocks and the effects of weathering processes (He et al., 2020; Ivanišević et al., 2023), subsequent changes (abrasion, attrition, dissolution, sorting, and deposition) during transport and sedimentation processes (Garzanti et al., 2009; Parlak et al., 2022; Rahman et al., 2022), and the impacts of hydrodynamic conditions (Jin et al., 2019; Lai et al., 2023; Singh & Basu, 2022). When studying transported sediments in a river load, it is necessary to determine their physical and chemical properties and mineral content, whether from clay, silt, or sand minerals, as these sediments will eventually be deposited, adding organic and mineral matter to the land where they are deposited (Al-Shihmani, 2022; Caracciolo, 2020). Most of the soil minerals found in sediments play an important role in agriculture: They contain many important elements that can maintain or increase the productivity of agricultural lands. They also serve as indicators for assessing soil fertility and chemistry (Al-Shammary et al., 2023a, 2025; Mirzaei et al., 2023; Xiong et al., 2022; Xu et al., 2023). Studying the type of soil-generating rocks and determining their mineral content in terms of quality and quantity is useful for informing the effective management of soils from an agricultural perspective. Variations in the mineral composition of the parent materials from which these soils and sediments are derived significantly influence the physicochemical properties and overall soil fertility (Al-Shammary et al., 2023b; Castillo et al., 2021; Taher et al., 2019).

Numerous studies have assessed the mineral content of sediments from the Tigris and Euphrates rivers. These investigations have identified a range of minerals, including light and heavy sand and clay minerals (Ahmed & Mohammed, 2022; Garzanti et al., 2016). Most studies have focused on the geological origin and formation of these sediments (Al-Kaaby & Albadran, 2020; Du et al., 2022), in addition to determining their importance in industrial and developmental fields (Habib & Abd Burghal, 2023). However, few mineral studies have focused on agricultural applications, especially in the fields of soil chemistry and minerals. Taking into consideration the climatic and hydrological changes in recent years, climate change, specifically the reduction in rainfall, has resulted in decreased water erosion and increased wind erosion due to desertification. This shift has significant implications for the quantity and quality of river load sediments. Additionally, human activities, such as the construction of dams and reservoirs along river courses, further influence sediment dynamics by blocking and sorting sediments, thereby altering the overall sediment content in the river load (Al-Shihmani, 2022; Guo, 2024). More research is needed, especially on lands subject to sedimentation processes, such as the basins of the Tigris and Euphrates rivers (Al Baghdady & Alabadi, 2021; Al-Shammary et al., 2024).

Mineral mature sediments develop through various processes, the most significant of which is chemical and physical weathering (Guyot et al., 2007). Mineral maturity refers to the ratio of stable minerals, such as quartz and zircon, to unstable minerals, such as feldspar and lithic fragments, within a sedimentary deposit. This maturity reflects the origin and evolution of sand minerals, providing insight into the prevailing climate and environmental conditions experienced by the sediments over time, in addition to the transport distance and recycling intensity (Olivarius et al., 2022). As sediments are transported, their maturity typically increases as unstable minerals are gradually eroded or dissolved, leaving behind more stable minerals, such as quartz. A high proportion of quartz is often an indicator of mineral maturity among the light minerals in sand sediments (Gheith et al., 2021).

Mature sediments tend to contain mostly stable minerals and display a narrower range of mineral types than immature sediments, which can include both stable and unstable minerals (Garzanti, 2017).

In agriculture, particularly in soil chemistry and fertility, immature sediments are valued for their ability to form new minerals that benefit plant growth (Kaletova et al., 2022). Additionally, chemical maturity is often considered more important than physical maturity due to its critical role in plant nutrition and soil fertility.

The current study aims to determine the quality and quantity of heavy and light sand minerals in the sediment transported in the riverbed load of the Tigris and Euphrates rivers within the Iraqi sedimentary plain. The findings of this study are fundamental for understanding the chemical properties of sediments. This may improve some properties of agricultural soil when sediments are added or deposited on it. Recent climatic changes have led to water scarcity and altered the hydrological conditions of the Tigris and Euphrates Rivers, for example, dam construction, the emergence of river islands, and the development of meanders along the course of both rivers. All of these factors have affected the quantity and quality of the riverine sediment load, which may impact the soil properties where these sediments are deposited in the near future.

## Materials and methods

### Sampling collection

**Fig. 1:**
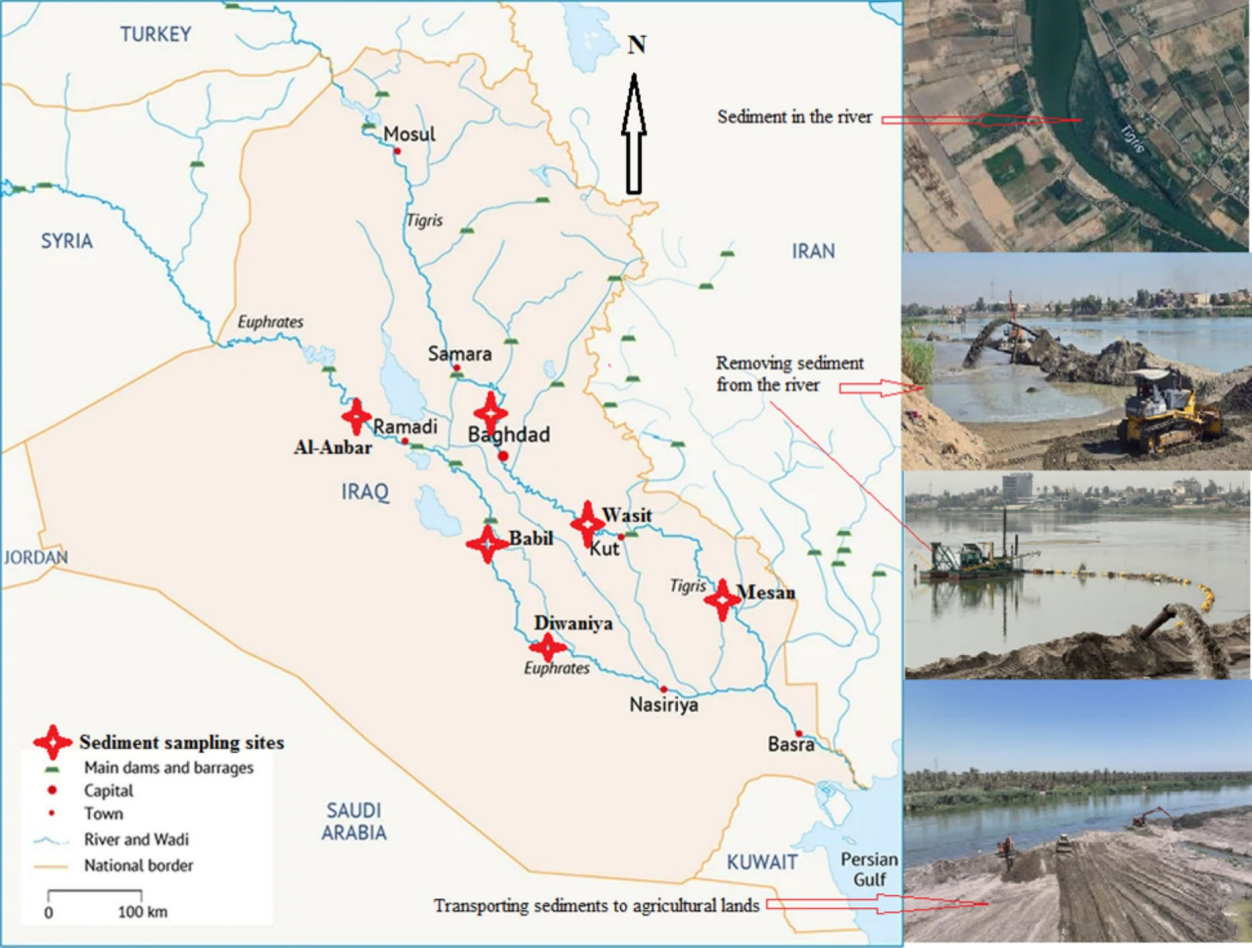
Location of sample collection sites from Tigris and Euphrates rivers

The Tigris and Euphrates Rivers begin in Turkey and flow southward into Syria before entering northern Iraq. This area is characterised by undulating terrain and steep slopes, which increase the river current velocity. However, as the rivers reach the current study area, which represents the alluvial plain, the slope and undulation of the land decrease. This reduction in gradient slows the flow of the river load, initiating sedimentation processes as the rivers progress through the alluvial plain in Iraq. The Tigris River sequentially flows through the governorates of Baghdad, Wasit, and Maysan. Similarly, the Euphrates River traverses the governorates of Al-Anbar, Babil, and Diwaniya, in that order. Six sites were identified for the study: three on the Tigris River and three on the Euphrates River, distributed across the aforementioned governorates. The distance between sites on each river is more than 100 km. Variation in riverbed sediment characteristics requires distances of more than 100 km between sites, especially when slope conditions are similar. Eight separate sub-sites were identified at each of the six studied locations, separated by several kilometres, based on the presence of sediments near the riverbank. Samples from these sub-sites were collected and combined into a single composite sample. The samples were collected from surface river sediments accumulated near the riverbanks during the summer of 2022, when river water levels were low due to water rationing resulting from water scarcity caused by climate change. Most sediments appear near the riverbank when the water level is low, making them easier to collect. An auger was used to collect sediment samples from a depth of 0–30 cm. Sediment samples were collected from the side of the river where sedimentation occurred. A total of 1 kg of composite sample was collected from each site and transported to the laboratory in a plastic container. Sampling sites were marked on a map along the riverbank in the designated governorates of the Tigris and Euphrates rivers, according to the coordinates, Baghdad (33°28′30.1″ N, 44°17′59.3″ E), Wasit (32°37′51.0″ N, 45°23′17.2″ E), and Maysan (31°53′04.6″ N, 46°56′39.0″ E). Al-Anbar (33°48′59.9″ N, 42°43′59.6″ E), Babil (32°41′24.7″ N, 44°15′23.1″ E), and Diwaniya (31°52′07.1″ N, 44°56′12.6″ E), as shown in Fig. 1.

After the samples were collected, they were dried, and their particles were separated using a wooden rod and passed through a 2-mm sieve. All sediment particles were smaller than 2 mm, except for some undecomposed organic materials, due to the distance travelled by the sediment and the low intensity of the water currents. The resulting sediment was used for physical and chemical analyses.

### Sediment physical and chemical assessments

The particle size distribution of the sediment was determined using the pipette method (Gee & Or, 2002). The sediment pH and electrical conductivity were measured using a WTW Cond 7110 device with a 1:1 (w/v) solution. The sediment water extract was prepared according to international methods (Al-Shammary et al., 2022; Page et al., 1982). The carbonate content was determined using the Nelson’s (1983) method, and the cation exchange capacity was assessed following the procedure reported by Papanicolau (1976). Additionally, the organic matter was determined using the Walkley–Black method, as published in Page et al. (1982).

### Mineral analysis

To identify the light and heavy sand minerals, sand particles were separated from clay and silt by wet sieving through a 50-µm sieve. After the sieving process, the sand particles were washed with distilled water to remove any remaining clay. The sand particles were then oven-dried at 60 °C and washed with acetone. Five grams of sand particles were placed in a separation funnel containing bromoform liquid (CHBr_3_) with a specific gravity of 2.89 and separated according to previously reported methods (Rocks, 1974; Tucker, 1988). The percentages of light and heavy mineral fractions in the sand particles were calculated after the separated samples were washed with acetone, dried, and accurately weighed. Heavy sand particles were scattered on a glass slide and fixed with Canada balsam, an adhesive with a refractive index of 1.54. A similar treatment was applied to the light sand minerals. The heavy and light sand minerals were analysed separately using polarised light microscopy (PLM) according to previously reported methods (Hall, 1978; Nesse, 2000). The percentage of each mineral was calculated using a point-counting device for 250–300 sand grains per slide, according to Black et al. (1965).

The mineralogical maturity index of light sand minerals was calculated using the following equation (Pettijohn, 1975), which is particularly suitable for sedimentary deposits in Iraqi regions:

$$Minera\log ical\;Maturity\;Index\;(MMI)\:=\frac{Quartz}{Feldspar+Rock\;Fragments}\times100$$

The mineralogical maturity index for heavy sand minerals was determined using the equilateral triangle method. This involved distributing the sum of the percentages of stable, unstable, and phyllosilicate minerals within the triangle to assess the overall mineral maturity.

## Results and discussion

### Sediment assessment

The importance of studying the chemical properties of sediments lies in their influence on the properties of agricultural soils on which these sediments are deposited. High pH values (pH > 7.8) can affect the availability of most trace elements and phosphorus in the soil, thereby reducing its fertility. Additionally, an increase in organic matter (OM) content in the sediments enhances soil fertility. An increase in cation exchange capacity (CEC) also indicates a greater ability of the sediments to improve soil fertility. Furthermore, an increase in calcium carbonate content helps prevent sodium accumulation and regulates soil pH. In this study, the sediments were found to be alkaline, with pH values ranging from 7.4 to 8.2 (Table 1). Most Iraqi sediments are alkaline because of the predominance of carbonate minerals (Rostaminia et al., 2020) and generally low organic matter content. The observed pH values fall within the agriculturally acceptable limits, albeit with some constraints.

**Table 1:** Chemical and physical properties of soil

Clay %	Silt %	Sand %	CEC cmol kg^−1^	CaCO_3_%	OM %	EC ds m^**−**1^	pH	Location	
10	16	74	8.65	25.50	1.38	1.13	8.1	Tigris	Baghdad
12	25	63	13.09	31.43	2.11	1.25	7.8		Wasit
15	29	56	15.12	27.84	2.82	1.34	7.4		Mesan
14	18	68	9.43	33.21	1.21	1.20	8.2	Euphrates	Al-Anbar
16	20	64	18.54	26.64	3.10	1.31	7.9		Babel
18	23	59	17.83	27.12	2.78	1.44	7.5		Diwaniya
10	16	56	8.65	25.50	1.21	1.13	7.4	min	
18	29	74	18.54	33.21	3.10	1.44	8.2	max	
14	22	64	13.78	28.62	2.23	1.28	7.8	mean	

Electrical conductivity (EC) values were low, ranging from 1.13 to 1.44 ds m^−1^. The sediments of the Tigris and Euphrates Rivers are largely salt-free due to continuous leaching associated with river water level fluctuations. These fluctuations result from hydraulic structures (regulators and dams) and the management of water quotas and releases according to distribution plans, causing the water height in the channels to vary. The coarse sediment texture (Table 1) facilitates the removal of salt. There was a slight increase in EC values with distance along the rivers, attributed to return flows from urban areas (Mohammed et al., 2021). The EC of sediments from the Euphrates River was also higher than that of the Tigris River, reflecting differences in water sources and the nature of the terrains through which each river passes, which affect their physical and chemical properties as well as transport and sedimentation processes. Notably, these low EC values indicate that removing river sediments from their depositional sites and applying them to surrounding lands, either directly or through irrigation with sediment-laden water, is unlikely to cause a significant increase in soil salinity.

The amount of organic matter (OM) was low at most sites, ranging from 1.21 to 3.10% (Table 1). This was due to the lack of vegetation cover in Iraqi lands due to high temperatures and low rainfall, which contributes to the increased oxidation of organic matter (Nassif et al., 2023). Although the organic matter values in the sediments are indeed low, they remain acceptable given the conditions of Iraqi soils.

Carbonate minerals in the bed sediments of the river load of the Tigris and Euphrates rivers ranged from 25.5 to 33.2%. These high values are due to several carbonate sources. The first source is the transport of carbonate minerals by the river load in the form of sediments from the highlands in the northern regions, which are then deposited with the river load sediments. The second source is the capillary ascent of carbonate from groundwater and its deposition in the presence of calcium and carbonate ions upon saturation. The third source is the deposition of carbonates from irrigation water after the combination of calcium and bicarbonate ions (Delver, 1962). The values of carbonate minerals in the sediments are somewhat high, but they prevent the formation of sodic soils in agricultural lands.

The cationic exchange capacity (CEC) of the bed sediment ranged from 8.65 to 18.54 cmol kg^−1^. CEC is an indicator of the density of negative charges for fine sediment particles and OM through a direct relationship (Mishra et al., 2022). These CEC values were relatively low due to the low percentage of both OM and clay particles. However, CEC values falling within the low to medium range can be improved by incorporating mineral or organic colloids if these sediments are applied to agricultural lands.

Sand particles predominated over other sediment particle types, accounting for 56–74%. This is because the sediments were transported as part of the bed load, moving through saltation, rolling, and sliding mechanisms due to their greater specific weight and large size compared to the rest of the sediment particles (Rentier & Cammeraat, 2022). The proportion of sand particles decreased with distance downstream along the Tigris and Euphrates Rivers due to the deposition of some of these particles during the transportation process. This was a result of low-intensity river water currents caused by hydrological conditions affecting the rivers.

Clay particles accounted for 10–18% of the sediment composition (Table 1). These relatively low values are attributed to the fact that most clay particles remain in suspension within the river load (Lamb et al., 2020). However, the proportion of clay particles increased with distance downstream along both the Tigris and Euphrates Rivers.

The higher proportion of sand particles in the sediments, when added to lands with elevated clay content, can enhance the physical properties of those soils, thereby improving their overall quality.

### Mineral study

Current research trends increasingly focus on rock fertilisers and rock fragments as slow-release nutrient sources. This involves studying and determining the content of rock fragments and sediments that contain essential elements for enhancing agricultural land and promoting sustainability (Van Straaten, 2006). Sand minerals are recognised as slow-release nutrient reservoirs for plants. Notably, feldspar, mica, and olivine serve as potassium sources within sand minerals. Additionally, various sand minerals can supply other vital nutrients, including silicon, iron, zinc, copper, nickel, sodium, calcium, and magnesium (Mohammed et al., 2014; Swoboda et al., 2022).

Light sand minerals constituted 95.6–96.8% of the sediments, whereas heavy sand minerals made up 3.2–4.4% at the study sites (Table 2). The large difference between the proportion of heavy and light sand minerals may have been due to the nature of the rocks that generated these deposits in the source area, in addition to the effect of weathering on the sediments, whether in the source area, during transportation, or after sedimentation (Al Baghdady & Alabadi, 2021).

**Table 2:** The weight ratio of sand minerals (heavy to light)

Location		Sample weight (g)	Light mineral weight (g)	Light minerals (%)	Heavy mineral weight (g)	Heavy minerals (%)
Baghdad	Tigris	5	4.82	96.4	0.18	3.6
Wasit		5	4.79	95.8	0.21	4.2
Mesan		5	4.78	95.6	0.22	4.4
AL-Anbar	Euphrates	5	4.84	96.8	0.16	3.2
Babel		5	4.81	96.2	0.19	3.8
Diwaniya		5	4.80	96.0	0.20	4.0

The proportion of light sand minerals decreased as the distance along the rivers increased, whereas that of heavy sand minerals increased. This may be due to the saltation, rolling, and sliding of the sediments, and the fact that particles of light sand minerals may have a higher tendency for fragmentation than heavy sand minerals. Therefore, a greater proportion of light sand minerals would have become suspended in the river load as the distance along the river increased.

Table 3 shows the light sand minerals in the bed sediments of the Tigris and Euphrates rivers. The following minerals were identified: quartz (polycrystalline quartz and monocrystalline quartz), feldspar (potash feldspar microcline, potash feldspar orthoclase, and plagioclase feldspar), and fragmented rock minerals (carbonate rock fragments, chert rock fragments, mudstone rock fragments, evaporates, igneous rock fragments, and metamorphic rock fragments). The light sand minerals also contained minor quantities of other minerals.

**Table 3:** Percentages of light minerals

Light components		Sampling site					
		Baghdad	Wasit	Mesan	AL-Anbar	Babel	Diwaniya
Quartz	Monocrystalline quartz	44.6	35.7	30.5	33.2	32.2	30.1
	Polycrystalline quartz	2.7	2.3	1.6	2.6	2.4	2.2
Feldspars	Potash feldspar microcline	2.5	2.6	2.9	3.0	3.1	3.4
	Potash feldspar orthoclase	3.6	2.2	4.7	3.1	3.6	3.5
	Plagioclase feldspar	2.5	2.9	4.4	1.6	1.9	2.2
Rock fragments	Carbonate rock fragments	21.4	31.6	33.6	27.0	28.3	30.4
	Chert rock fragments	7.6	5.8	6.2	8.8	7.8	8.1
	Mudstone rock fragments	4.7	5.5	4.2	6.1	6.1	6.5
	Evaporites (gypsum)	2.1	5.4	5.7	6.6	6.6	6.4
	Igneous rock fragment	2.4	1.6	1.5	2.6	2.5	2.6
	Metamorphic rock Fragments	3.7	3.2	3.3	3.3	3.1	3.2
Coated grains by clay		1.5	0.9	1.1	1.8	1.3	0.8
Others		0.7	0.3	0.3	0.3	1.1	0.6

**Fig. 2:**
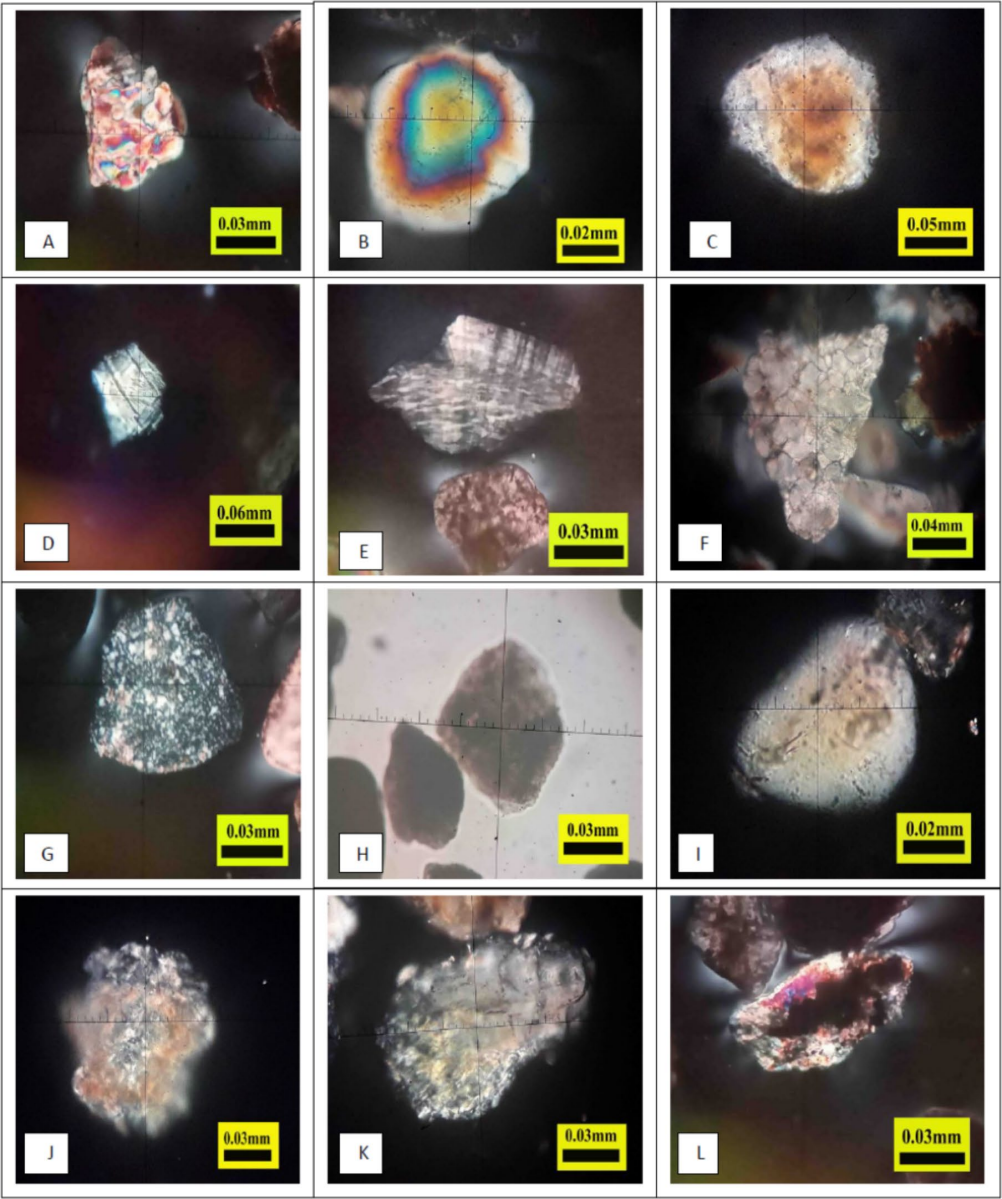
Polarised light microscopy images of some light sand minerals. **A** Polycrystalline quartz; **B** monocrystalline quartz; **C** feldspar orthoclase; **D** feldspar plagioclase; **E** feldspar microcline; **F** carbonate rock fragments; **G** chert; **H** mudstone; **I** gypsum; **J** igneous rock; **K** metamorphic rock; **L** grains coated by clay

The proportions of monocrystalline and polycrystalline quartz ranged from 30.1 to 44.6% and 1.6 to 2.7%, respectively, across all study sites. Quartz is a light mineral, and its high percentage in the samples may be attributed to its high resistance to physical weathering (Götze et al., 2021). The proportions of both forms of quartz decreased as the distance along the rivers increased. This decline is due to the increased sedimentation with distance, the inability of quartz to fully resist weathering during water transport, and the reduced intensity of the water currents. Quartz was examined using polarised light microscopy, which revealed that the polycrystalline quartz crystals were medium-sized, semi-angular in shape, and exhibited angular extinction (Fig. 2A). The monocrystalline quartz crystals were medium in size, semi-round in shape, and displayed both angular and parallel extinction (Fig. 2B).

The presence of quartz in sediments is beneficial for agriculture. Although quartz is more resistant to physical weathering than many other minerals, it is susceptible to chemical weathering, forming products such as dissolved silica, which can be absorbed by certain plants to enhance stem strength, particularly in crops like rice (Lanning, 1963).

Potash feldspar microcline accounted for 2.5 to 3.4% (Table 3), with higher percentages in the sediments of the Euphrates River compared to the Tigris River, and with increasing percentages in both rivers further downstream. Potash feldspar orthoclase accounted for 2.2 to 4.7%, and plagioclase feldspar for 1.6 to 4.4%, with greater percentages in the Tigris River than the Euphrates River. This may be due to the distinct hydrological and environmental characteristics of the Tigris and Euphrates rivers (Salman & Essa, 2020). The proportion of potash feldspar increased with increasing distance along both rivers; this mineral is an important natural source of potassium, contributing to sediment and soil fertility (Ciceri et al., 2019). The potash feldspar orthoclase particles were white and pink, with moderate roundness and curvature (Fig. 2C). The plagioclase feldspar particles were white and exhibited twinning, rounded, and medium-sized (Fig. 2D). The potash feldspar microcline particles were semi-angular in shape and medium in size (Fig. 2E).

Carbonate rock fragments ranged from 21.4 to 33.6%, which is a higher percentage than that of other light sand minerals. This was due to the high percentage of carbonate rocks in the source area (Fayyadh & Sindi, 2021; Sissakian & Saeed, 2012). The percentage of carbonate rock fragments increased with distance in both rivers, mainly because of their ability to dominate both fine and coarse sand particles. This mineral is not easily destroyed during transportation and therefore remains present in the river load (Dolatabadi et al., 2020). The carbonate rock fragments were observed under polarised light microscopy, revealing angular to semi-angular shapes, large to medium sizes, a white pearly colour, and a vitreous-like lustre (Fig. 2F). Chert rock fragments ranged from 5.8 to 8.8%, with a higher percentage in the Euphrates River than in the Tigris River, due to the greater abundance of this mineral in the Euphrates River source area. Chert has important effects on sediments and agricultural soils, especially on the physical and chemical properties of soils derived from this mineral. The presence of chert affects the clay content, pH, CEC, and the base saturation ratio of the soils and sediments (Taher et al., 2019). The chert rock fragments examined under polarised light microscopy were large to medium in size and semi-circular to semi-angular in shape because of the weathering resistance of this mineral (Fig. 2G). Mudstone rock fragments ranged from 4.2 to 6.5%, with higher percentages in the Euphrates River than in the Tigris River. This may be due to variations in the effect of chemical weathering in the source regions. Chemical weathering alters pH levels, increasing the dissolution of mudstone rock fragments within sediments transported by water erosion. This is because mudstone is a mixture of clay minerals, quartz, mica, feldspar, and iron oxides (Su et al., 2020). The mudstone rock fragments, observed under polarised light microscopy, had a brown colour and semi-angular shape (Fig. 2H). The proportion of mudstone rock fragments decreased with increasing distance along the rivers, especially along the Euphrates River (Table 3). Evaporites (gypsum) ranged from 2.1 to 6.6%, with a higher percentage in the Euphrates River than in the Tigris River. This may be attributed to the higher proportion of gypsum minerals along the Euphrates river in addition to the source areas compared to the Tigris River (Al-Hetty et al., 2021). The proportion of evaporites increased along the Tigris River, possibly due to the increase in the EC of the river water. The gypsum minerals were examined under polarised light microscopy and appeared white with medium to small circular particles (Fig. 2I). Igneous rock fragments ranged from 1.5 to 2.6%, with higher percentages in the Euphrates River than in the Tigris River. Their percentage decreased as the distance along the Tigris River increased, reflecting the variation in the mineral content of the source area. These fragments, examined under polarised light microscopy, appeared white to pink, medium-sized, and semi-angular to semi-circular shapes (Fig. 2J). Metamorphic rock fragments ranged from 3.1 to 3.7%, with similar proportions in both rivers. These were examined by polarised light microscopy (Fig. 2K). Grains coated by clay minerals ranged from 0.8 to 1.8% and were also examined by polarised light microscopy (Fig. 2L).

From the analysis, it is evident that increasing the percentage of carbonate rock fragments, along with gypsum in light sand minerals, can enhance the reclamation of sodic soils with high sodium content. Additionally, if present, these materials can help reduce soil acidity, as carbonate rock fragments contribute to shifting the soil reaction towards neutrality.

As shown in Table 4, the heavy sand mineral fractions in the Tigris and Euphrates River sediments consisted of opaque minerals, chlorite, orthopyroxene, clinopyroxene, hornblende, glaucophane, micas (biotite, muscovite), zircon, tourmaline, rutile, garnet, epidote, staurolite, and kyanite.

**Table 4:** Percentages of heavy minerals

Heavy minerals		Sampling sites					
		Baghdad	Wasit	Mesan	AL-Anbar	Babel	Diwaniya
Opaques		42.6	40.1	34.2	36.1	34.9	32.4
Chlorite		6.4	9.6	10.1	6.9	7.1	7.5
Pyroxene group	Orthopyroxene	4.9	2.2	2.0	4.2	3.2	2.1
	Clinopyroxene	4.7	4.5	4.6	6.4	5.9	5.6
Amphibole group	Hornblende	4.3	7.2	8.1	8.6	6.2	6.8
	Glaucophane	0.2	1.2	1.5	1.9	2.5	0.3
Mica group	Biotite	5.3	4.7	5.2	4.5	7.5	7.1
	Muscovite	7.7	4.8	6.6	3.8	4.8	7.3
Zircon		3.8	2.4	4.3	6.3	6.6	5.9
Tourmaline		3.5	4.1	5.7	5.8	3.9	5.6
Rutile		2.9	2.7	2.9	2.3	2.9	3.5
Garnet		4.6	5.9	3.4	3.6	4.1	5.5
Epidote		5.8	6.4	6.1	4.2	5.9	5.2
Staurolite		1.4	1.1	2.5	2.6	1.8	2.4
Kyanite		1.5	2.4	1.6	2.1	1.6	2.2
Others		0.4	0.7	1.2	0.7	1.1	0.6

**Fig. 3:**
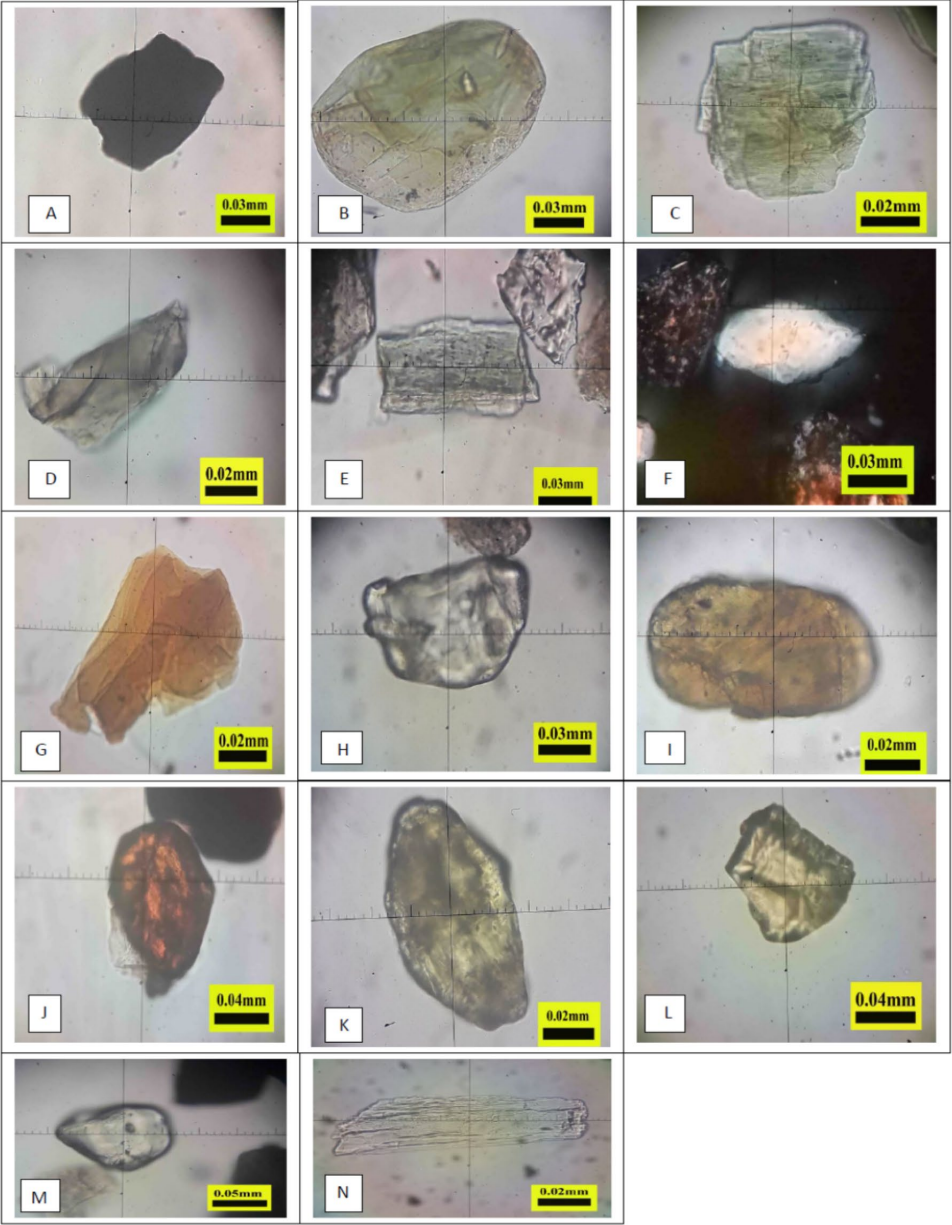
Polarised light microscope images of some heavy sand minerals. **A** opaque minerals; **B** chlorite; **C** orthopyroxene; **D** hornblende; **E** glaucophane; **F** muscovite; **G** biotite; **H** zircon; **I** tourmaline; **J** rutile; **K** epidote; **L** staurolite; **M** garnet; **N** kyanite

Opaque minerals ranged from 32.4 to 42.6%, with a higher percentage in the Tigris River than in the Euphrates River and a decreasing trend as the distance along the rivers increased. The higher content of quartz and opaque minerals may indicate that the sediment is affected by weathering processes, while the presence of calcite and dolomite in the sediment indicates a reduction in the intensity of weathering (Juita, 2020). The morphological characteristics of these minerals were examined by polarised light microscopy, revealing semi-angular to semi-circular, medium-sized granules with an opaque colour and metallic lustre (Fig. 3A). The opaque minerals primarily consist of iron oxides, sulphur, and nickel, which may undergo chemical weathering, resulting in the formation of dissolved ions, such as iron, that can be utilised by plants. Chlorite ranged from 6.4 to 10.1%, with a higher percentage in the Tigris River than in the Euphrates River. The chlorite percentage increased with distance along the rivers because of its ability to coat sand minerals, such as quartz. The multiple sources of chlorite minerals vary depending on the formation environment (Worden et al., 2020). The chlorite minerals appeared as medium circular particles with semi-rounded edges, a light green colour, and vitreous lustre (Fig. 3B). Chlorite is a silicate mineral that contains magnesium, iron, and aluminium, and may also contain calcium, nickel, manganese, potassium, sodium, and chromium. It may undergo chemical weathering, which releases some dissolved ions. The pyroxene group minerals, orthopyroxene and clinopyroxene, were identified, ranging from 2.0 to 4.9% and 4.5 to 6.4%, respectively. These were present in higher percentages in the Euphrates River sediments than in the Tigris River and decreased with increasing distance along the Euphrates River. The abundance of pyroxene group minerals indicates that the sediment is a significant source of Mg (Prasetyo et al., 2004), suggesting that it has undergone intense physical weathering with limited chemical weathering (Füchtbauer & Schmincke, 1974). Under polarised light microscopy, orthopyroxene appeared pale green with a vitreous lustre and semi-round shape (Fig. 3C). The amphibole group minerals, hornblende and glaucophane, were also identified. Hornblende ranged from 4.3 to 8.6%, and its percentage increased with distance along the Tigris River. Glaucophane levels ranged from 0.2 to 2.5% and increased with increasing distance along the Tigris River. Minerals, such as plagioclase, amphibole, and pyroxene, are easily weathered and contribute macronutrients, such as Ca, Mg, Na, and K, to the sediments and soils (Bastaman & Pramuji, 2009). Understanding the mineral composition of sediment offers valuable insights into its fertility potential, as the nutrient content in minerals contributes to the sustainability of sediment and soil fertility (Esmaeilian et al., 2023; Nasir et al., 2021). Under polarised light microscopy, hornblende exhibited a prismatic shape and vitreous lustre (Fig. 3D), while glaucophane appeared colourless, with a vitreous lustre, elongated shape, sharp edges, and large- to medium-sized particles (Fig. 3E).

The mica group minerals, biotite and muscovite, were identified. Biotite ranged from 4.5 to 7.5%, while muscovite accounted for 3.8 to 7.7%. The proportions of these minerals increased with increasing distance along the Euphrates River, likely due to their lightweight and lamellar structure (Al Shamary et al., 2022), which facilitates transport with the river load, even under low-intensity current. These minerals are present in all sediment and soil particles and can release potassium into the soil through chemical weathering (Basak, 2019). Under polarised light microscopy, muscovite appeared white and flaky with medium to large particle sizes (Fig. 3F), while biotite was light brown with a semi-angular lamellar shape and medium to small particles (Fig. 3G). Zircon minerals ranged from 2.4 to 6.6%, with higher percentages in the Euphrates than in the Tigris. This increase reflects more intense weathering because zircon is highly resistant to weathering. Additionally, zircon minerals can be used to prepare plant growth extracts that are used as growth regulators to increase the production of some crops (Amirov et al., 2019). Under polarised light microscopy, zircon appeared colourless and sub-angular in shape (Fig. 3H). Tourmaline minerals ranged from 3.5 to 5.8%, with the percentage increasing with distance along the Tigris River, reflecting their resistance to weathering. Tourmaline can be used in agriculture for the preparation of growth regulators that have a role in promoting plant growth as effective and safe growth stimuli for plants (Huang & Lee, 2022). The minerals were characterised by polarised light microscopy as honey-coloured with a semi-round shape and medium size (Fig. 3I). Rutile minerals ranged from 2.3 to 3.5%, with their percentage increasing with distance along the Euphrates River. Nanoscale rutile particles play a key role in photosynthesis enhancement and plant genetic modification (Mariz-Ponte et al., 2022). Under polarised light microscopy, rutile appeared deep red, with medium to large particles and high relief (Fig. 3J). Garnet minerals ranged from 3.4 to 5.9%. These were observed under polarised light microscopy as colourless, semi-rounded, and exhibiting high relief (Fig. 3M). Epidote minerals ranged from 4.2 to 6.4%, with higher values in the Tigris than in the Euphrates. Epidotes play a role in the release of basic cations, particularly calcium, into sediments and soils. With increasing chronological age, epidotes may contribute to the slow-reacting pool of calcium (Basdediós et al., 2022). Under polarised light microscopy, the epidote appeared light green, with a vitreous lustre and prismatic shape (Fig. 3K). Staurolite minerals ranged from 1.1 to 2.6%. These were identified by polarised light microscopy as yellowish, with a vitreous lustre and angular shape (Fig. 3L). Kyanite minerals ranged from 1.5 to 2.4% and were characterised by polarised light microscopy as colourless and prismatic (Fig. 3N). The polarised light microscopy results highlight the morphological diversity of the identified light and heavy sand minerals. The variation in shape, size, and form reflects the effect of physical weathering due to the transport of bed sediments by river load, which can lead to the erosion and fragmentation of some sand mineral edges. These differences are influenced by the specific weight of each mineral and the ability of the mineral to resist weathering. Silicate sand minerals provide several agronomic benefits, including improved soil structure, enhanced water retention and drainage, supply of essential nutrients to plants, regulation of soil pH, and, in some cases, natural resistance against pests.

The mineral maturity index values for light sand minerals, as shown in Table 5, ranged from 48.25 to 93.64%, with an average of 60.93%. These values were generally higher in the Tigris River than in the Euphrates River. This difference may be attributed to variations in both the quantitative and qualitative mineral composition in the source areas of the two rivers, which generate the sediments. Additionally, the differences in the type and intensity of weathering processes, whether occurring at the source, during river transport, or within the sedimentation zone, also play a significant role (Adama et al., 2024). The results suggest that the sediments of the Tigris River underwent more intense weathering processes during the successive transport and deposition stages, particularly within the bedload sediments of the river.

**Table 5:** Mineral maturity index of light sand minerals

MMI	Rock fragments	Feldspars	Quartz	Location	
%					
93.64	42.84	8.80	48.36	Tigris	Baghdad
62.49	53.75	7.79	38.46		Wasit
48.25	55.28	12.17	32.55		Mesan
57.62	55.58	7.86	36.56	Euphrates	Al-Anbar
54.91	55.74	8.81	35.45		Babel
48.69	58.03	9.22	32.75		Diwaniya
48.25	42.84	7.79	32.55	Min	
93.64	58.03	12.17	48.36	Max	
60.93	53.54	9.11	37.35	Mean	

Table 5 shows a decreasing trend in the mineral maturity index of light sand minerals with increasing distance along both the Tigris and Euphrates Rivers. In the Tigris River, the mineral maturity index within the Baghdad Governorate was 93.64%, but it decreased to 62.49% in the Wasit Governorate and to 48.25% in the Maysan Governorate. This decline is likely attributable to the increasing percentage of feldspar minerals and rock fragments at the expense of quartz minerals (Agbenyezi et al., 2022). A possible explanation for this change is the influence of small rivers and tributaries, particularly those entering the Tigris after Baghdad, which introduce sediments from adjacent Iranian territories. These sediments have a shorter transport path from the Tigris River’s source in Turkey to Baghdad (Al-Shihmani et al., 2024). Additionally, the differences in the mineral content of sediments from these two sources contribute to the observed variation.

Regarding the Euphrates River, a similar decline in the mineral maturity index with distance is observed. In the Al-Anbar Governorate, the index was 57.62%, decreasing to 54.91% in Babil Governorate and 48.69% in the Diwaniyah Governorate. This decline can also be attributed to the increasing presence of feldspar minerals and rock fragments at the expense of quartz. Moreover, western winds may contribute by transporting sediments from the Western Desert, which have a different mineral composition from those carried by the river’s bedload (Muhs, 2004). This variation in sediment input affects the mineral content of the river load as it moves downstream. The qualitative and quantitative differences in mineral content between the river’s source area and the Western Desert further contribute to the reduction in the mineral maturity index (Kwankam et al., 2023).

**Fig. 4:**
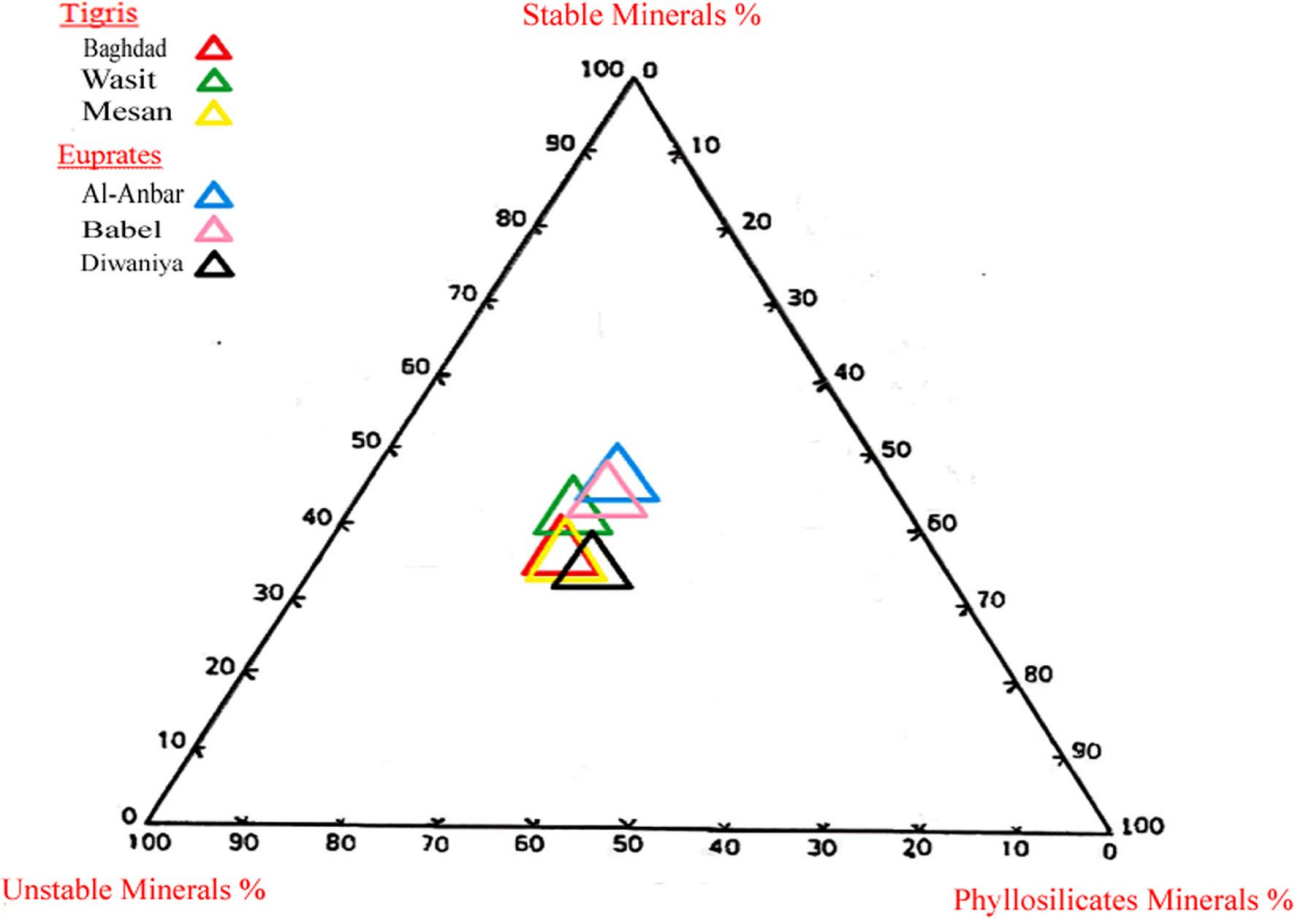
Mineral maturity index for heavy sand minerals

Table 6 and Fig. 4 show that the mineral maturity index for heavy sand minerals is dominated by unstable minerals, with values ranging from 33.95 to 45.02% and an average of 39.70%. Phyllosilicate minerals are the second most abundant group, ranging from 63.05 to 38.02%, with an average of 34.97%. Stable minerals ranked third, with percentages between 23.24 and 28.87% and an average of 25.33%.

**Table 6:** Percentage of phyllosilicates minerals, unstable minerals, and stable minerals

Stable minerals	Stable minerals	Unstable minerals	Phyllosilicates minerals	Location	
%					
23.24		38.87	37.89	Tigris	Baghdad
23.39		40.56	36.05		Wasit
23.26		38.72	38.02		Mesan
27.93		45.02	27.05	Euphrates	Al-Anbar
25.31		41.07	33.62		Babel
28.87		33.95	37.18		Diwaniya
23.24		33.95	36.05	Min	
28.87		45.02	38.02	Max	
25.33		39.70	34.97	Mean

According to the general rule, the higher the values for stable minerals, the greater the increase in mineral maturity, as indicated by their position towards the top of the triangular plot. The maturity type, physical or chemical, is determined by the distance of the projected values from the top of the triangle for phyllosilicate and unstable minerals. A greater distance from the top of phyllosilicate suggests a trend towards physical maturity, whereas a greater distance from the top for unstable minerals indicates chemical maturity (Selley, 1976).

The results suggest that the overall maturity of the studied sediments is low, primarily due to the low proportion of stable minerals. Despite the close distribution of values, the maturity trend leans more towards physical maturity. This may be attributed to several factors: The sediments are relatively newly formed, the transport distance by river load was short, and the high speed of transport—possibly driven by floods—limited the degree of chemical alteration. Furthermore, the source rocks contained higher proportions of unstable and phyllosilicate minerals than the stable ones. The arid climate of the deposition area likely also inhibited chemical weathering processes, further contributing to the lower sediment maturity.

The findings from this study indicate that these sediments, whether deposited naturally or transported by humans, can be beneficial when added to agricultural lands with poor soil characteristics. These sediments improve the conditions of heavy clay soils by improving soil structure, drainage, and water retention. Chemically and mineralogically, they help mitigate the risk of sodicity and regulate soil pH. Additionally, these sediments serve as an important reservoir for essential nutrients such as potassium, magnesium, silicon, aluminium, manganese, calcium, nickel, and iron, thereby improving agricultural sustainability in lands that lack these vital components.

### Limitations of the current study and future investigations

This study provides a broad examination of sand minerals, offering valuable insights into their general properties and occurrences. However, several limitations inherent to the current research highlight areas for deeper exploration. A critical gap lies in the detailed analysis of individual sand minerals. Future studies should delve into the specific properties of these minerals (XRF, ICP-OES, SEM–EDS), with a particular emphasis on their chemical compositions, especially for those minerals that occur in significant proportions. Such investigations could yield a more nuanced understanding of the materials and their potential applications.

Another important area for future research is the relationship between sand minerals and environmental pollution. This remains an underexplored aspect of sedimentology, despite its relevance to ecosystem health. Understanding how these minerals contribute to or mitigate pollution in various ecosystems could inform strategies for environmental management and remediation. In parallel, more comprehensive studies tracking mineral deposits from their source areas downstream to river mouths are needed. Such research would illuminate the processes of mineral distribution and transformation, offering insights into their behaviour and accumulation in different environments.

The potential agricultural applications of sand minerals also warrant further investigation. Future research should evaluate the effects of incorporating contemporary mineral deposits into agricultural soils, particularly in relation to their suitability for specific crop needs. This line of inquiry could provide guidance on optimizing land use and improving soil fertility. Similarly, the construction and engineering sectors could benefit from studies that assess the significance of these deposits as raw materials. Detailed evaluations of their mechanical properties and durability could inform industry practices and material selection.

From a methodological perspective, integrating mathematical models to estimate sediment deposition rates represents a promising avenue for enhancing predictive accuracy. By applying quantitative approaches, researchers could better anticipate changes in sediment dynamics, aiding both scientific understanding and practical applications. Finally, attention should be directed to marshes and natural water bodies linked to river systems. These environments play critical roles in sediment retention and blockage, yet their influence on sediment dynamics remains poorly understood. Exploring their impact could provide valuable insights into sediment transport and ecosystem services. In addressing these limitations and pursuing these suggested areas, future research can significantly expand our understanding of sand minerals, their applications, and their interactions with natural and anthropogenic systems.

## Conclusions

The importance of conducting mineral studies on river sediments, particularly those accumulated in rivers and added to agricultural lands, whether transported by human activities or deposited as a result of irrigation with sediment-laden water, cannot be overstated. These studies are crucial for assessing the properties of soils affected by sediments, as they help determine the potential for managing these agricultural lands to ensure optimal and sustainable productivity. Mineralogical and chemical studies on the quantity and quality of sediment in the river bedload of the Tigris and Euphrates Rivers, especially concerning possible agricultural applications, are currently lacking. In recent years, these sediments have been affected by environmental and climatic changes. This study examined the variation in the quantity and quality of heavy and light sand minerals between the Tigris and Euphrates Rivers, as well as the variation along each river. Differences in some physical, chemical, and mineralogical properties of sediments were observed between the two rivers. Heavy and light sand minerals were identified using a polarising light microscope, with traces of physical erosion observed on the surface of some minerals because of the transport process in the river bedload. These sediments are newly formed and exhibit low mineral maturity with a more physical maturity trend. It is recommended that these sediments, whether deposited naturally or transported by humans to agricultural lands during river dredging, be used to improve the properties of heavy clay soils, regulate soil pH, prevent sodicity, and add an important stock of sand minerals that provide the soil with some essential elements. Such applications would contribute to the achievement of agricultural sustainability.

## Data Availability

No datasets were generated or analysed during the current study.
